# Effects of Probiotics and Lactoferrin on Necrotizing Enterocolitis in Preterm Infants

**DOI:** 10.7759/cureus.18256

**Published:** 2021-09-24

**Authors:** Saleh Al-Alaiyan, Najlaa Abdulaziz, Albatoul Alkohlani, Sana O Almairi, Fahad Al Hazzani, Abdulaziz Binmanee, Areej Alfattani

**Affiliations:** 1 Pediatrics/Neonatal-Perinatal Medicine, King Faisal Specialist Hospital & Research Centre, Riyadh, SAU; 2 Pediatrics, Alfaisal University, Riyadh, SAU; 3 Pediatrics, King Faisal Specialist Hospital & Research Centre, Riyadh, SAU; 4 Pediatrics, Alfaisal University College of Medicine, Riyadh, SAU; 5 Biostatistics and Epidemiology, King Faisal Specialist Hospital & Research Centre, Riyadh, SAU

**Keywords:** neonates, necrotizing enterocolitis, probiotics, lactoferrin, premature infants

## Abstract

Background

Necrotizing enterocolitis (NEC) is a leading cause of morbidity and mortality in neonates. Despite intensive research, the etiology and pathophysiology of NEC is still obscure. Evidence from recent studies and meta-analyses showed a significant role of probiotics as a prophylactic measure in reducing NEC, sepsis, and mortality. However, obstacles against the generalization of the results still remain. The aim of the study was to evaluate the role of prophylactic administration of probiotics and lactoferrin in reducing the rate of NEC in preterm infants.

Methods

In this retrospective cohort study, all medical records of infants born with a birth weight of 1,500 g and less who were born between 2012 and 2017 were reviewed. The enrolled infants were divided into two groups: group 1 included infants born between January 2012 and August 2014, a period before probiotics were started in our unit, and group 2 included infants born between January 2014 and December 2017 after starting probiotics and lactoferrin. Multiple variables were collected including maternal data, neonatal data, and risk factors for NEC.

Results

Medical records of 284 infants who met our inclusion criteria were reviewed. Of the 284 infants, 134 were in group 1 and 150 infants were in group 2. There were no significant statistical differences between group 1 and group 2 in neonatal and maternal demographic data and clinical data. Of 134 infants who received probiotics and lactoferrin, 11 developed NEC, while 26 of the 150 infants in group 2 developed NEC, and the difference was statistically significant (p = 0.023).

Conclusion

Probiotics and lactoferrin given orally to very low birth weight preterm infants were associated with a decreased rate of NEC.

## Introduction

Necrotizing enterocolitis (NEC) remains a leading cause of gastrointestinal-related morbidity and mortality, predominantly affecting premature infants in the neonatal intensive care unit (NICU) [1]. The disease is known to manifest as inflammatory necrosis of the bowel leading to a sequela of morbidities, including neurodevelopmental impairment and gastrointestinal dysfunction [2]. Decades of meticulous research on the complex disease suggested a multifactorial pathophysiology, with one of the most important factors being that premature intestines have a markedly increased expression of TLR4, which, when activated by microbial colonization, leads to intestinal injury, decreased mucosal repair, and microbial translocation, all of which trigger the vasoconstriction and necrosis of intestinal tissue in NEC [3].

Several endogenous and environmental risk factors have been identified to potentially trigger the events leading to bowel compromise, some of which cannot be controlled by the NICU caregivers, such as birth weight, gestational age, and any possible genetic variation [4]. The type of feeding is an important controllable factor in the development of NEC. Studies of the gut microbiota in formula-fed infants showed the absence of bifidobacteria, which are present in breastfed healthy infants and are believed to promote intestinal health in neonates [5]. Respiratory distress syndrome (RDS) was found to be significant in increasing the risk of NEC, while the introduction of surfactant was deemed protective due to the improved respiratory function decreasing the ischemia [6]. Preeclampsia-related intrauterine growth restriction (IUGR) is also suggested to be one of the factors increasing the risk of NEC [7]. Another identified risk is hypotension requiring the administration of inotropes, especially in the first week after birth [6]. Both early- and late-onset sepsis were found to increase the incidence of NEC in several studies [8].

The overall survival rates of preterm infants have notably increased over the years due to advancements in medical care, but with that the rate of infants at risk of many morbidities, including NEC, and the burden on healthcare systems has also increased [9]. Given the fulminant clinical course of the disease and the risk of severe complications that are associated with it, an urgent need for preventive measures that ensure both efficacy and safety is rising, especially considering the insufficiency of currently used interventions for the management of NEC [10,11]. Preventative interventions have mainly targeted modifications in feeding protocols and antibiotic administration given their influence on NEC’s pathophysiological factors [4].

Probiotics are “live microorganisms which when administered in adequate amounts confer a health benefit on the host” [12]. They have increasingly been studied and utilized as a potential preventive strategy for NEC due to their anti-inflammatory effect on the intestinal epithelium [13,14]. Current evidence from the most recent clinical trials, systemic reviews, and meta-analysis demonstrates a major contribution of the prophylactic use of probiotics in the reduction of the risk of developing NEC and the subsequent morbidity and mortality in preterm infants [14,11]. Still, the data available lack in identifying clear advantages in the safety and efficacy of the use of probiotics [8].

Lactoferrin is a natural glycoprotein found in human colostrum and, in smaller concentrations, in human milk. Given its role in innate immunity and broad-spectrum antimicrobial effect, lactoferrin administration has been found to provide significant protection against neonatal sepsis and a synergistic effect to probiotics against NEC, especially in premature infants that were strictly formula fed [15].

In August 2014, our NICU adopted a policy of routine prophylactic administration of probiotics and lactoferrin in preterm infants as a preventative measure for NEC. The aim of this retrospective cohort study was to assess whether the routine supplementation of probiotics and lactoferrin was associated with a decrease in the rate of NEC in premature infants.

## Materials and methods

Study design and setting

This is a single-center retrospective cohort study review of newborns admitted to the tertiary-level NICU at King Faisal Specialist Hospital & Research Centre, Riyadh, Kingdom of Saudi Arabia. Our hospital serves as a national tertiary/quaternary care and referral center and is one of the leading healthcare institutions in the Middle East. Ours is a level IV NICU that provides highly specialized care for a patient population from all over the country. We annually admit 50-60 neonates with a gestational age of ≤32 weeks [16].

Study period

Medical records of patients born between January 1, 2012, and December 31, 2017, were reviewed.

Eligibility criteria

Eligible neonates comprised all inborn and outborn neonates with a birth weight of ≤1,500 g and less than 35 weeks of gestational age who were fed and tolerated feeds for five days within the allocated study period. Neonates with major congenital anomalies and neonates who died early in their hospital course were excluded from the study.

Probiotic administration protocol

Routine probiotic administration protocol was introduced in our NICU in December 2014. Probiotics and lactoferrin were usually initiated on the second day of life for all neonates with birth weight ≤ 1,500 g and were continued daily until a diagnosis of NEC was made or the patient reached 35 weeks’ corrected age. We used the probiotic Lactobacillus GG (Dicoflor, Dicofarm, Rome, Italy) in this study with a dosage of three drops/day for <1,000 g infants and five drops/day for ≥1,000 g infants. We also used bovine lactoferrin (BLF 100, Dicofarm) sachets with a dosage of one sachets/day for <1,000 g infants and two sachets /day for ≥1,000 g infants.

Feeding practices

All infants were fed either expressed breast milk (EBM), formula milk, or both as per the feeding protocol of the Saudi Neonatology Society [17]. Although preferred, the majority of infants could not be exclusively breastfed due to small gestational age, lack of breast milk, or lack of population awareness.

Outcomes and measures

All eligible patients’ medical records were reviewed from the period of admission to discharge/death for definite or suspected NEC. NEC was assessed and scored according to Bell’s staging, and all the radiographic images were reviewed by certified pediatric radiologists [18,19]. Sepsis was defined as having signs and symptoms of infection with or without accompanying bacteremia.

Data collection and management

All medical records of infants born with the aforementioned inclusion criteria within the allocated study period were reviewed. The enrolled infants were divided into two groups: group 1 included infants born between January 2012 and August 2014 (pre-probiotics group) and group 2 included infants born between December 2014 and December 2017 (probiotics group).

Multiple variables were collected, including maternal data: age, use of antenatal steroids, premature rupture of membrane (PROM), and mode of delivery. Neonatal data included gestational age (GA), gender, birth weight, and birth location (outborn vs. inborn). Risk factors included IUGR, type of feeding (formula vs. human milk), need for blood transfusion, metabolic acidosis, hypotension, need for patent ductus arteriosus (PDA) treatment, respiratory support, prolonged initial antibiotics, umbilical venous and arterial catheterization, and positive blood or urine cultures.

Statistical analysis

After data management and coding, the SAS software (SAS Institute, Cary NC) was used for statistical analysis. Descriptive statistics were calculated. Outcome analysis was performed in the light of the identified risk factors. Chi-square tests along with Fisher’s exact test were used to explore the relationships between dependent variables. Univariate analysis of the categorical data was performed using logistic regression models for parametric distributions and relevant risk factors. A level of significance of <0.05 was considered to assess the relative risk (RR) with 95% confidence interval.

## Results

Medical records of 284 infants who met our inclusion criteria were reviewed. Of the 284 infants, 150 neonates in group 1 did not receive probiotic or lactoferrin supplementation, whereas all 134 in group 2 received probiotics and lactoferrin as per our protocol.

A comparison between the two groups in Table 1 showed no statistically significant differences in maternal age (30.29 ± 6.5 years vs 28.93 ± 8.9 years; a p-value of 0.14), antenatal steroids (p = 0.72), IUGR (p = 0.52), and singleton versus multiple pregnancy (p = 0.23). PROM was more in group 2 and statistically significant (p = 0.005).

**Table 1:** Maternal and neonatal demographic characteristics in the two groups IUGR, intrauterine growth restriction; PROM, premature ruptured of membranes; SVD, spontaneous vaginal delivery; BWT, birth weight; GA, gestational age

Variables	Probiotic and lactoferrin (n=134)	No probiotic and lactoferrin (n=150)	p-Value
Maternal age	30.29 ± 6.5	28.93 ± 8.9	0.14
IUGR, n (%)	46 (44.7%)	57 (55.3%)	0.52
Antenatal steroids, n (%)	98 (48.3%)	105 (51.7%)	0.72
PROM, n (%)	15 (30.6%)	34 (69.4%)	0.005
Singleton, n (%)	77 (44.5%)	96 (55.5%)	0.23
SVD, n (%)	27 (46.6%)	31 (53.4%)	0.82
BWT (grams)	1,087 ± 281	1148 ± 264	0.59
GA (weeks)	29.60 ± 2.60	29.66 ± 2.81	0.90
Sex (male %)	63 (49.6%)	64 (50.4%)	0.46
Outborn, n (%)	1 (0.75%)	14 (9%)	0.001

The neonatal demographic characteristics showed no statistically significant differences between groups 1 and 2 in birth weight (1,087 ± 281 g vs 1,148 ± 264 g; p = 0.59), gestational age (29.60 ± 2.60 vs 29.66 ± 2.81; p = 0.90), and gender (male % of 49.6% vs 50.4%; p = 0.46). The majority of infants who did not receive probiotics and lactoferrin were outborn (p = 0.001).

In the present study, we found that the rate of NEC was more among infants who did not receive probiotics and lactoferrin (8.2% 17.3%) and the difference was statistically significant (p = 0.023); however, there were no statistically significant difference between the two groups in the stages of NEC (p = 0.15) and the age when NEC was diagnosed (23.82 ± 13.2 days vs 19.2 ± 12.7 days; p = 0.32) (Table 2). We also found no statistically significant difference between the two groups in risk factors of developing NEC that included blood transfusion (p = 0.18), blood acidosis (p = 0.61), use of inotropes (p = 0.49), sepsis (p = 0.83), prolonged initial antibiotics (p = 0.22), PDA treatment (p = 0.90), feeding (EBM, formula, and mixed) (p = 0.078), respiratory support (invasive vs. non-invasive) (p = 0.23), and use of umbilical venous catheter (p = 0.10) and umbilical arterial catheter (p = 0.63).

**Table 2:** NEC and risk factors of developing NEC in the two groups NEC, necrotizing enterocolitis; EBM, expressed breast milk; PDA, patent ductus arteriosus; UVC, umbilical venous catheter; UAC, umbilical arterial catheter; UTI, urinary tract infection

Variables	Probiotic and lactoferrin (n=134)	No probiotic and lactoferrin (n=150)	p-Value
NEC, n (%)	11 (8.2%)	26 (17.3%)	0.023
Age at diagnosis of NEC (days)	23.82 ± 13.2	19.2 ± 12.7	0.32
Blood transfusion, n (%)	53 (42.7%)	71 (57.3%)	0.18
Acidosis, n (%)	34 (44.7%)	42 (55.3%)	0.61
Inotropes use, n (%)	31 (43.7%)	40 (56.3%)	0.49
Feeding – EBM, n (%)	18 (46.2%)	21 (53.8%)	0.078
Prolonged initial antibiotics, n (%)	54 (55.1%)	44 (44.9%)	0.22
PDA treatment, n (%)	108 (80.6%)	120(80%)	0.9
Respiratory support invasive, n (%)	62 (44%)	79 (56%)	0.23
UVC, n (%)	118 (49.2%)	119 (50.2%)	0.10
UAC, n (%)	38 (50%)	38 (50%)	0.63
Sepsis, n (%)	19 (48.7%)	20 (51.3%)	0.83
Age at sepsis (days)	18.43 ± 10.53	19.73 ± 19.83	0.78
UTI, n (%)	7 (46.7%)	8 (53.3%)	0.96

## Discussion

NEC remains one of the most common emergencies in neonatology, resulting in significant mortality and long-term morbidity [2]. The exact pathogenesis of NEC is still unclear, but multiple risk factors were described including prematurity, enteral feeding, and the use of indomethacin for treating PDA [2,20]. Many previous studies have evaluated the potential protective impact of probiotics on NEC, which demonstrated some contradictory results and the need for further research in the area.

In this single-center retrospective cohort study evaluating the effect of routine use of probiotics for preventing NEC, we identified a significant reduction in the rate of NEC following the implementation of our probiotics administration protocol. Consistent with our hypothesis, we found that the rate of NEC fell remarkably (from 17.3% to 8.2%; p = 0.023) in our sample of 284 neonates. This statistically significant risk reduction is also consistent with significant NEC risk reductions reported by recent systematic reviews and meta-analyses. In a meta-analysis, which included 23 randomized controlled trials (RCTs) and 4,686 infants, authors found that Lactobacillus contributed to a significant reduction in NEC incidence in preterm infants and death [21]. Moreover, they found no significant difference in the incidence of sepsis between the Lactobacillus and placebo groups. In another systematic review that included 30 articles, authors concluded that probiotic supplement could significantly reduce the risk of stage II-III NEC and death [22].

Although evidence for the effectiveness of lactoferrin as a preventive measure for NEC in preterm infants is still not well established, a recent systematic review and meta-analysis that included nine RCTs involving 1,34 patients showed that prophylactic lactoferrin could significantly reduce the incidence of NEC and all culture-proven late-onset sepsis in preterm infants [23].

In the context of our study, NEC risk reduction was irrespective of any evaluated demographic variables except among outborn infants and those who were born to mothers with a history of PROM in the group who did not receive probiotics and lactoferrin.

Although breastfeeding has been reported to have a protective impact on NEC [20], our analysis did not demonstrate any significant contribution of EBM administration. However, it is still unclear if the reported protective effect is related to breast milk itself or the avoidance of formula milk [24]. Nonetheless, this needs to be cautiously considered in the context of the present study given the fact that EBM from donors is strictly limited in our facility due to religious and cultural restrictions. On the other hand, prolonged initial antibiotics therapy was believed to contribute to NEC due to decreased bacterial diversity and promotion of potential pathogens growth [25]. However, our study did not demonstrate a significant association between prolonged initial antibiotics therapy and NEC rates in our sample. Furthermore, we expected NEC rates to be affected by variations in mode of delivery, need for antenatal steroid or PDA treatment, and diagnosis of sepsis given the reported association in previous studies [20,24,26-27]. Nonetheless, these associations were not established in our analysis.

To our knowledge, this is the first study in Saudi Arabia to evaluate the protective effect of routine probiotics administration on NEC rates in premature infants. Another strength of this study is the effort to control variation in our patient population by including multiple potential confounders in our analysis. Moreover, the specialized care in our facility ensured early and compliant administration of probiotics as per our protocol.

Given the retrospective and observational nature of this study, causality between the introduction of probiotics and the observed reduction in NEC risk cannot be supported. Other unmeasured confounders might underlie the observed overall risk reduction of NEC. The present study is also limited by the small, single-centered patient database of a highly specialized NICU, which limits the generalizability of the results. Finally, considering the time duration of this study, changes in the clinical practice in our NICU may have contributed to the overall NEC risk reduction which we did not measure.

Nevertheless, the present study demonstrates a statistically significant decline in NEC rates after the administration of probiotics and lactoferrin, which is comparable to previous studies. This would serve as beneficial comparative data for other researchers on the prophylactic effect of probiotics. This study also provides a base for generating hypotheses for future studies and evaluations of similar practices in the region and worldwide aiming collectively at improving the clinical outcome of patients with NEC, which remains a challenge till today.

## Conclusions

We conclude that probiotics and lactoferrin given orally to very low birth weight preterm infants were associated with a decreased rate of NEC. Furthermore, we found no decrease in the rate of sepsis among infants who received probiotics and lactoferrin and controls.
